# Interventions to reduce dependency in personal activities of daily living in community-dwelling adults who use homecare services: protocol for a systematic review

**DOI:** 10.1186/2046-4053-2-49

**Published:** 2013-07-02

**Authors:** Phillip J Whitehead, Avril ER Drummond, Marion F Walker, Ruth H Parry

**Affiliations:** 1 Division of Rehabilitation and Ageing, University of Nottingham, B Floor, The Medical School, Queens Medical Centre, Nottingham NG7 2UH, UK; 2 Faculty of Medicine and Health Sciences, University of Nottingham, A Floor, The Medical School, Queens Medical Centre, Nottingham NG7 2HA, UK

**Keywords:** Homecare services, Personal activities of daily living, Re-ablement, Restorative care, Occupational therapy

## Abstract

**Background:**

There is a growing demand for services whereby individuals receive assistance from care workers for personal care within the home. This has led to the development of re-ablement or restorative homecare services that provide time-limited input aimed at reducing dependency in personal activities of daily living, and preventing or delaying the need for further homecare support. However, little is currently known about how such interventions are configured, or how they may affect individuals’ ability to carry out personal care independently.

**Methods/Design:**

We will seek to identify studies that compare an intervention designed to reduce dependency in personal activities of daily living with routine input or usual care as the control. We will include randomised controlled trials, nonrandomised controlled trials, and controlled before and after studies. We will also include interrupted time series studies.

We shall search electronic databases in addition to searching for ongoing and unpublished studies, and where appropriate will contact key authors. Two reviewers will independently screen articles for inclusion; will assess risk of bias using quality assessment tools; and will carry out data extraction using pre-prepared forms. Any disagreements, at any stage, will be resolved by discussion and the involvement of a third reviewer if needed. We will produce a narrative summary of the results. A meta-analysis will be conducted if sufficient data are available of appropriate quality and comparability.

**Discussion:**

The findings from this review will inform future practice within homecare re-ablement services; will inform policy decisions about the structure, organisation and content of such services; and will identify areas where further research is warranted.

**Trial registration:**

This review protocol has been registered on the PROSPERO database (CRD42013004163).

## Background

Personal care is important for independence and includes tasks such as washing, dressing and feeding. People experiencing difficulties with these tasks may receive homecare or domiciliary care services in order to enable them to remain living safely at home [1]. Such services usually involve a paid carer visiting the person to provide assistance with the tasks with which they are having difficulty. These services have commonly adopted a ‘doing for’ approach [2] whereby carers have taken over tasks that the person is no longer able to manage [3]. These services may be delivered by public or private health or social care organisations [4].

In the developed world, the demand for homecare services is increasing due to the combination of an ageing population, an increased emphasis on community-based care, and a reduction in the capacity for informal care provision within the family unit [5,6]. As demand for homecare services has increased, so too have the costs associated with care provision. Furthermore, there may be a shortfall in the number of workers to supply the amount of care which will be required in the coming decades [7]. The growing demand for homecare services combined with the increasing costs of providing this care have led to a focus on developing preventative services that aim to prevent or delay the need for care and support [8,9]. Such schemes – termed homecare re-ablement in the UK, and restorative home care in the USA, New Zealand and Australia [1,10] – aim to provide time-limited intensive input to facilitate users’ confidence and ability to carry out their own care independently, thereby reducing the need for ongoing homecare services [11].

However, little is currently known about how interventions are configured, their optimum timing and intensity, their effects on individuals, and the carryover of any effects [12,13]. The quality of the current evidence is also currently unclear.

### Description of the condition

Impaired ability to carry out activities of daily living may be related to frailty, or a disability, due to one or more chronic conditions [14]. Temporary impairment may also result from an event or injury (for example, hip fracture [15]). The term ‘activities of daily living’ encompasses the range of everyday tasks which individuals require for independent living [16] and may also be referred to as functional independence. Personal activities of daily living encompass those aspects that specifically relate to the management of personal care and hygiene needs, also often referred to as self-care. These aspects include, but are not limited to: washing, dressing, bathing/showering, feeding, toileting, management of continence, transfers, and basic mobility. Although older people may be more likely to experience difficulties in managing personal activities of daily living due to multiple morbidities, impaired ability to perform these activities does not exclusively affect older people; people of all ages with temporary or permanent disabilities may be affected.

### Description of the intervention

We intend to review interventions that have been provided to individuals who are receiving homecare services where the aim is to reduce their dependency in personal activities of daily living. The core components of the intervention are likely to include: repeated assessment and monitoring of performance in activities of daily living; training and repeated practice of activities of daily living tasks; education about self-management and associated techniques; assistance to build social support networks; and the provision of assistive devices (equipment) and home adaptations. Goal setting may also be a feature of the intervention in which individuals are assisted to achieve independence through a graded and staged process.

These interventions may consist of re-ablement or restorative care packages or programmes that are delivered as an alternative to standard care. However, because it is still unclear whether re-ablement and restorative care programmes are interventions in their own right or whether they comprise multiple interventions [12,17], and because these services are relatively new [4], we intend to review any intervention that has been delivered to adult users of homecare services, in their own home, with the aim of reducing dependency in personal activities of daily living. This may or may not be delivered as part of a re-ablement or restorative programme. The intervention may be delivered uniprofessionally or multiprofessionally, and may be delivered by qualified and/or nonqualified staff. The presence of the care workers may be used for confidence building and they may often accompany an individual whilst they complete the task themselves rather than the traditional approach of doing the activity for the user [18].

Occupational therapists have specialist skills in providing interventions targeted at performance in activities of daily living. These interventions have been shown to be effective at improving performance in activities of daily living in individuals in other contexts [15,19-22] and thus have the potential to be effective with a population of adult users of homecare services. However, the effect of occupational therapy involvement in homecare re-ablement services is currently unclear and has been highlighted by the UK Social Care Institute for Excellence as a priority for further research [23].

### Objectives

The objectives of this study are: to determine what interventions for adult users of homecare services, targeted at reducing dependency in personal activities of daily living, have been provided and evaluated; to determine the efficacy and effectiveness of these interventions on individuals’ dependency in activities of daily living; and to determine whether interventions involving occupational therapists differ in their effect on users’ performance in personal activities of daily living from those that do not involve occupational therapists.

## Methods/Design

### Criteria for considering studies for this review

#### Types of studies

We will include randomised controlled trials, nonrandomised controlled trials, and controlled before and after studies that compare an intervention designed to reduce dependency in personal activities of daily living with routine input or usual care as the control intervention. We will also include interrupted time series studies (where there is a clearly defined time point at which the intervention occurred and at least three data collection points before and after the intervention).

We will not include observational or qualitative studies.

#### Types of participants

Participants will include any individual, aged 18 years or older, living in a non-institutionalised home in the community, in receipt of homecare services. We define homecare as one or more weekly visit(s) from a paid carer (that is, not an unpaid relative or friend) to provide assistance with a personal activity of daily living. We distinguish this from home healthcare, which includes those services offered by qualified (registered or licensed) professionals including doctors, nurses and allied health professionals to individuals in their own homes. The homecare service may involve a home healthcare component (for example, nurse visits) but cannot be composed exclusively of home healthcare and must include routine assistance with personal activities of daily living by paid staff.

We will include studies that recruited people regardless of gender, ethnic group, medical diagnosis or multiple diagnoses, as long as they reside in the community and are in receipt of a homecare service.

We will exclude studies that focus on homecare services for an end-of-life care pathway.

#### Types of interventions

For the purpose of this review we are interested in studies of interventions that have the following features: provision of an intervention, delivered in or from the person’s home, that is primarily designed to reduce dependency in personal activities of daily living; and the intervention may comprise a single component (that is, profession specific or one-off visit) or multiple components (for example, a package provided by a multidisciplinary team).

We define usual care as a routine homecare service in which assistance with personal activities of daily living is provided, but where there is no intention to improve individuals’ performance with these activities.

The content of each intervention will be described narratively within the review.

#### Types of outcome measures

##### Primary outcomes

The two primary outcomes are: performance in personal activities of daily living (including washing, dressing, bathing/showering, feeding, toileting, management of continence, transfers, and basic mobility), an outcome that will take the form of an activities of daily living score (for example, Barthel Index); and a deterioration in the ability to perform activities of daily living (that is, a reduction in activities of daily living score), which will be a dichotomous outcome (for example, reduction in activities of daily living score or not).

##### Secondary outcomes

Individual outcomes will include: death; performance in extended activities of daily living (for example, shopping, outdoor mobility), measured using an extended or instrumental activities of daily living scale (for example, NEADL); number of participants admitted to hospital, residential or nursing care facilities; number of falls; participant mood/morale (measured using a questionnaire such as the General Health Questionnaire or Philadelphia Geriatric Morale Scale); health or social care related quality of life (for example, EQ5D or Adult Social Care Outcomes Toolkit); health economic outcomes; and caregiver strain/burden (for example, Caregiver Strain Index).

Service use outcomes (use of health and community services) will include: whether or not individuals are in receipt of homecare and the number of homecare support hours per week; participant and carer satisfaction with services; and healthcare provider satisfaction with the service.

#### Timing of outcome measures

We will group time points for outcome measures into three categories to represent short-term outcomes, medium-term outcomes, and long-term outcomes. These categories will be <6 months, 6 to 12 months, and >12 months, respectively.

### Search methods for identification of studies

#### Electronic searches

We will search the following electronic bibliographic databases: The Cochrane Central Register of Controlled Trials; MEDLINE (1948 to present); EMBASE (1980 to present); AMED (1985 to present); CINAHL (1982 to present); PsycINFO (1967 to present); Occupational Therapy database of systematic reviews and randomised controlled trials (OTseeker; 1980 to present); Physiotherapy Evidence Database (PEDro) (1929 to present); Web of Science (1990 to present); Center for International Rehabilitation Research Information and Exchange (CIRRIE; 1990 to present); and Applied Social Sciences Index and Abstracts (ASSIA).

The search strategy for Medline (Ovid) is shown in Appendix 1.

We will not restrict studies by language. We will include published conference abstracts.

#### Searching other resources

##### Ongoing research

We will identify ongoing research through the following databases: Current Controlled Trials (http://www.controlled-trials.com); Clinical Trials (http://www.ClinicalTrials.gov); and The Occupational Therapy Research Index and Dissertation Abstracts register.

##### Reference searching

We will examine the reference lists of all relevant papers for which we obtain the full text. We will also use the Science Citation Index Cited Reference Search for forward tracking of relevant papers.

##### Personal contact

We will contact key authors and researchers in the field to identify ongoing research and other sources of information.

### Data collection and analysis

#### Selection of studies

We will adopt a three-stage screening process. During the first stage, based upon the titles, one reviewer will exclude articles that are evidently not pertinent to the review. In the second stage, abstracts of all retained studies will be read. This will be completed independently and in duplicate by two reviewers. We will then obtain a paper copy of the full publication for every study that is potentially relevant. Two reviewers will then assess these independently and in duplicate. Disagreements at any stage of the selection process will be resolved through discussion by the reviewers, with the involvement of a third reviewer if necessary. The decisions will be recorded in writing.

#### Data extraction and management

Two reviewers will independently and in duplicate extract data from all included sources using pre-prepared and piloted data extraction forms. Extracted information will include: study methodology; study setting; study population and participant demographics and baseline characteristics; details of the intervention and control; recruitment and drop-out rates; outcome measurements and timing; and information for the assessment of risk of bias. These two reviewers will discuss any disagreements and will involve a third reviewer if required. We will seek additional information by contacting corresponding authors where necessary.

We will use Endnote X5 to manage the references. We will use Review Manager 5.2 to carry out the review and conduct the meta-analysis.

### Assessment of risk of bias in included studies

Two reviewers will independently assess the methodological quality of the included studies, using the risk of bias domain tool for studies with a separate control group (randomised controlled trials, nonrandomised controlled trials, and controlled before and after studies) developed by the Cochrane Effective Practice and Organisation of Care (EPOC) Group [24]. This assessment will cover sequence generation, allocation concealment, baseline characteristics, blinding of primary outcome assessment, completeness of outcome data, selective outcome reporting, and other potential sources of bias. Each of these factors will be explicitly rated and categorised as being at low, high or uncertain risk of bias.

To assess the risk of bias for interrupted time series we will use the seven standard criteria, as recommended by the Cochrane EPOC Group.

Disagreement between reviewers will be resolved by discussion and with the involvement of a third reviewer if necessary.

### Measures of treatment effect

For dichotomous outcomes (that is, death, reduction in activities of daily living score), we will express the intervention effect as a risk ratio with corresponding 95% confidence interval. For continuous outcomes (that is, activities of daily living score) we anticipate that different rating scales will be used, and so we will present the data as standardised mean differences with corresponding 95% confidence intervals.

### Unit of analysis issues

Where we include cluster trials that have randomised at group level rather than individual level, we will clearly label these in the narrative synthesis and meta-analysis. Where clustering has not been accounted for in the primary analysis, we will use the intracluster correlation coefficient. If the intracluster correlation coefficient is not available we will attempt to obtain it by contacting authors, or by imputing it with the assistance of a statistician.

In trials where a crossover design has been used, there would be a probable carryover effect from the first stage to the second. We will therefore only include data from the first phase of the study and we will clearly label any studies that have used a crossover design in the narrative synthesis and meta-analysis.

### Assessment of heterogeneity

We will include both randomised and nonrandomised studies, so we anticipate that we will encounter methodological heterogeneity. As the nature of the intervention may vary, we may also encounter clinical heterogeneity. Clinical heterogeneity will be examined using the proposed subgroup analyses, before reviewing comparison data. Methodological heterogeneity will be examined by comparing the results of randomised and nonrandomised studies. If there is any unexpected finding regarding clinical or methodological heterogeneity, then this will be discussed by all reviewers to seek consensus before proceeding with further analysis.

### Statistical heterogeneity

We will examine statistical heterogeneity by visually inspecting the forest plots and using the chi-squared (or *Q* statistic) and the *I*^2^ statistic. For each outcome, the decision to carry out a meta-analysis will be made by consensus among all authors.

### Assessment of publication bias

If appropriate and possible we will use funnel plots and assess funnel plot asymmetry.

### Data synthesis

A systematic narrative synthesis will be provided with information presented in the text and tables to summarise and explain the characteristics and findings of the included studies. The narrative synthesis will explore the relationship and findings both within and between the included studies, in line with the guidance from the Centre for Reviews and Dissemination [25].

If sufficient data are available and are of sufficient quality, a meta-analysis will be conducted. We will not combine the results of randomised and nonrandomised studies and these will be presented separately. For dichotomous outcomes we will use Peto odds ratios. For continuous data we will use a random effects model with an inverse variance method to generate the summary measures of effect in the form of the standardised mean difference.

For nonrandomised studies it is the exception rather than the rule to pool data [26] and we will only do so if studies are judged to be of sufficient quality and methodologically and clinically comparable. The decision to pool the data from nonrandomised studies will be by agreement of all reviewers and will follow the guidelines outlined in the Cochrane handbook. If we judge it appropriate to pool data from nonrandomised studies, we will use adjusted effect estimates, standard errors and the generic inverse variance method [26].

### Subgroup analysis

We will carry out a subgroup analysis of those interventions that have involved occupational therapists with those that have not, if there are sufficient data to do so.

Homecare services are provided to people of all ages, although some services may be restricted to older adults (which we will define as aged 65 or over). Therefore, if the data are available, we will carry out subgroup analyses for the following potential effect modifiers: interventions provided to older adults (aged 65 and over) compared with all adults; the intensity (how often) and the duration (how long) of the intervention; and whether the intervention was delivered uniprofessionally/multiprofessionally or by qualified/nonqualified staff.

### Sensitivity analysis

A sensitivity analysis will be carried out to explore the influence of study design. We will base this analysis on the method of randomisation or group allocation, adequacy of allocation concealment, presence of an intention-to-treat analysis, and blinding of the final outcome assessment.

## Discussion

The UK government has invested heavily in homecare re-ablement services as a means to facilitate increased independence at home and to reduce the costs of care provision [27,28]. Restorative care programmes are also being implemented in the USA, Australia, and New Zealand. Although the provision of these services is growing there is currently widespread variation in their organisation and content [13]. There is a lack of evidence regarding the outcomes for individual users and the mechanisms of the effects of interventions [12]. The optimum service delivery models are thus not clear. This review will seek to identify those interventions that have been delivered and evaluated to determine their effects. The findings from this review will therefore: inform future practice within re-ablement and restorative homecare services; inform policy decisions about the structure, organisation and content of such services; and identify areas where further research is warranted.

## Appendix 1: MEDLINE (Ovid) Search Strategy

### The search strategy below will be adapted for the other databases

1. home care services/

2. home health aides/

3. homemaker services/

4. homecare.tw.

5. (home$ adj1 (care$ or treat$ or help$)).tw.

6. (home$ adj2 (service$ or aid$)).tw.

7. domiciliary.tw.

8. 1 or 2 or 3 or 4 or 5 or 6 or 7

9. activities of daily living/

10. self care/

11. independent living/

12. (function$ adj1 (independ$ or abilit$)).tw.

13. (self adj1 care).tw.

14. (activit$ adj2 daily).tw.

15. (restorat$ adj2 (care$ or model$ or service$ or home$)).tw.

16. (re-able$ or reable$ or re-enablem$ or enablem$).tw.

17. goals/

18. (goal$ adj2 (set$ or treat$ or therap$)).tw.

19. 9 or 10 or 11 or 12 or 13 or 14 or 15 or 16 or 17 or 18

20. 8 and 19

21. randomized controlled trial.pt.

22. controlled clinical trial.pt.

23. (control$ adj2 trial).tw.

24. intervention studies/

25. experiment$.tw.

26. (time adj1 series).tw.

27. (pre test or pretest or posttest or post test).tw.

28. random allocation/

29. intervention?.tw.

30. evaluation studies/

31. comparative study.pt

32. 21 or 22 or 23 or 24 or 25 or 26 or 27 or 28 or 29 or 30 or 31

33. 21 and 32

34. nursing home/

35. 33 not 34

36. limit to adults

## Competing interests

The authors declare that they have no competing interests.

## Authors’ contributions

All authors contributed to the study design. PJW wrote the first draft of the protocol and led the design of the search strategy. All authors contributed to revisions of the manuscript and take responsibility for its content. All authors read and approved the final manuscript. AD, MW and RP are PW’s PhD supervisors.
